# Supplementary figures and images for: Suppression of Aedes aegypti by the sterile insect technique on Captiva Island, Florida, USA from 2020 to 2022
Source: PLoS Negl Trop Dis. 2025 Jul 11;19(7):e0013256. doi: 10.1371/journal.pntd.0013256 (PMC12310038; doi:10.1371/journal.pntd.0013256)

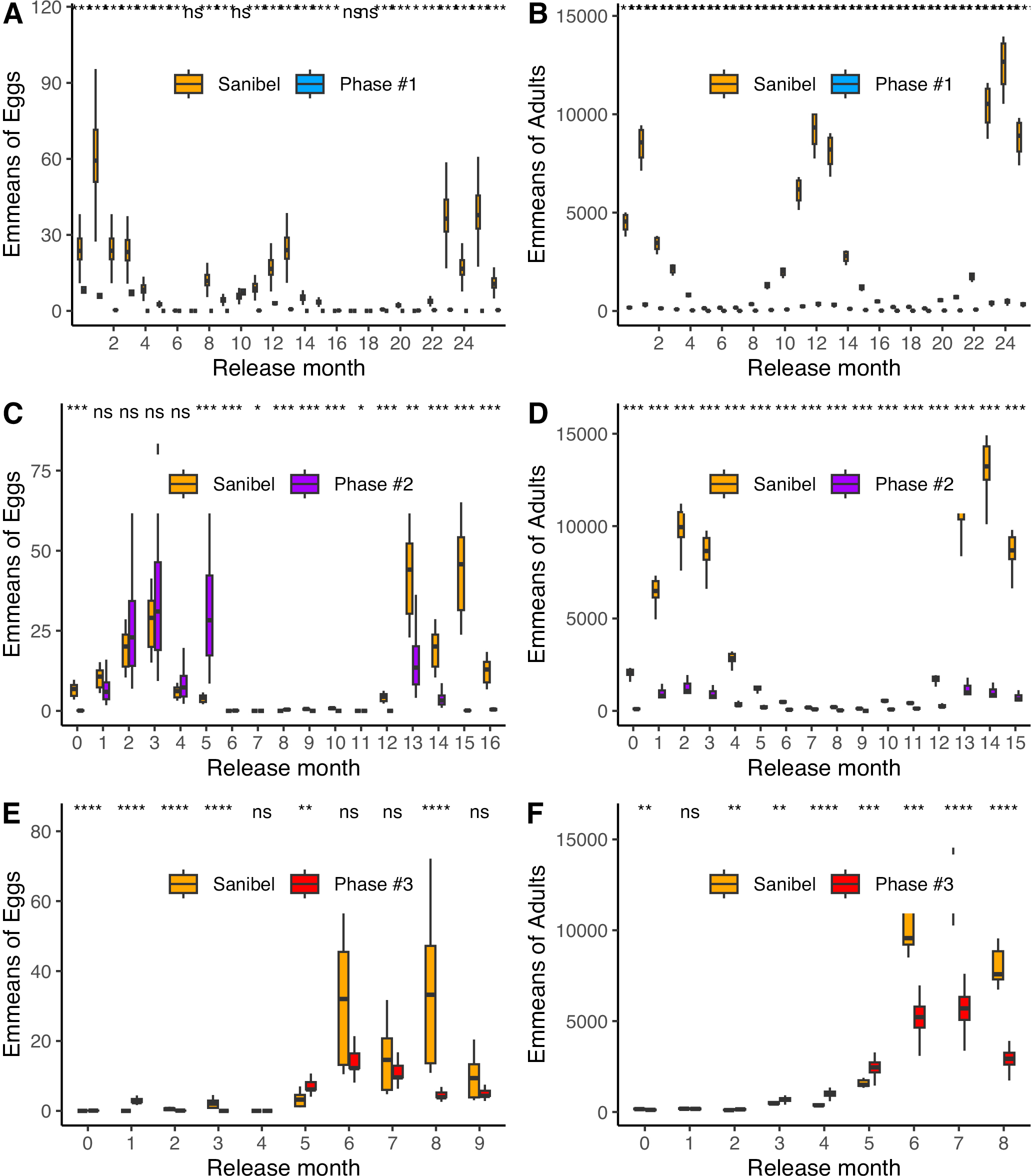

Supplement: S1 Fig — Each contrast was corrected for multiple comparisons and sampling times when the intervention and non-intervention areas were significantly different are marked with a *, whereas times when the intervention and non-intervention areas were not significantly different are marked with “ns”. (TIF) [file pntd.0013256.s001.tif]

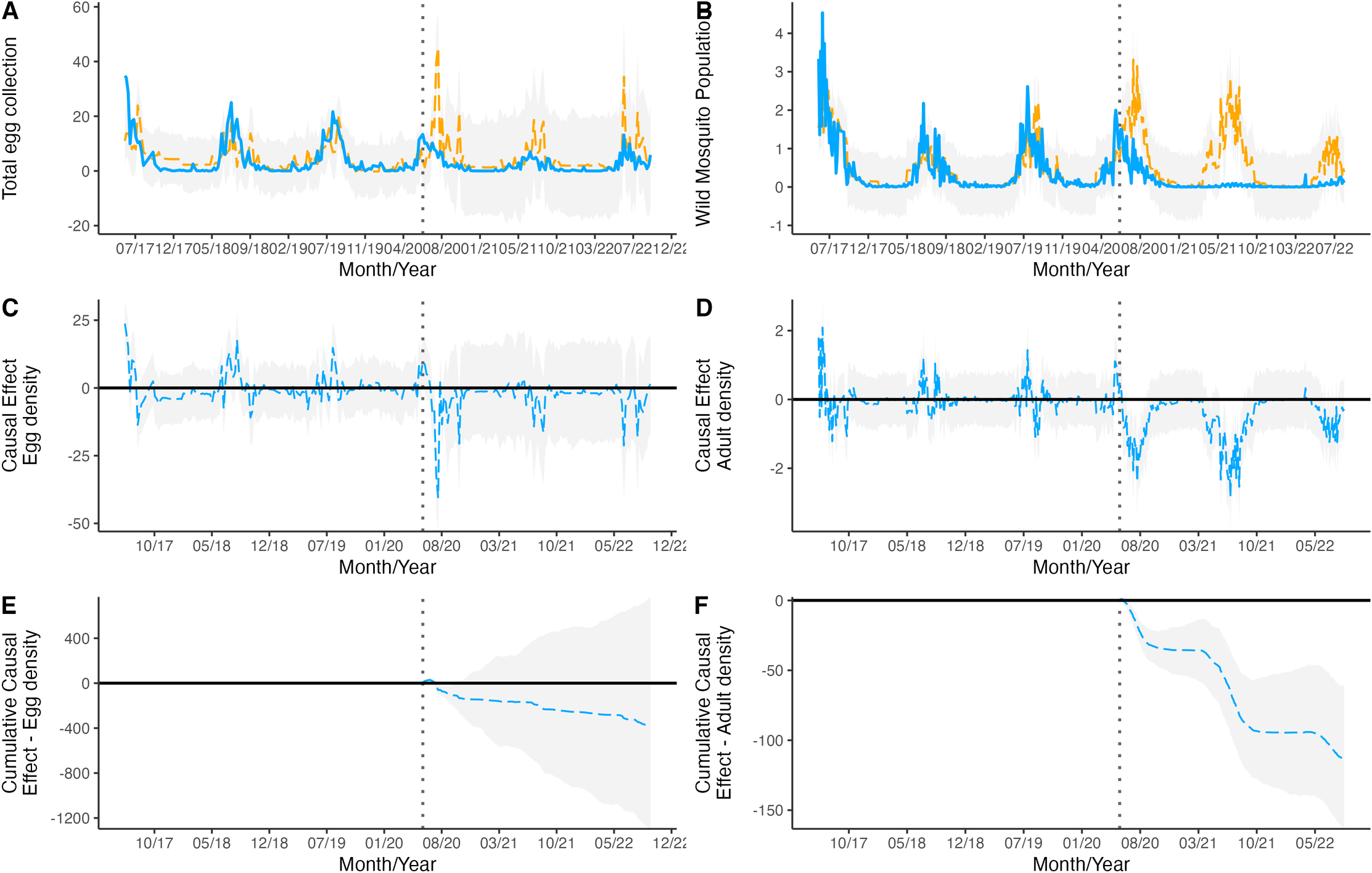

Supplement: S2 Fig — (TIF) [file pntd.0013256.s002.tif]

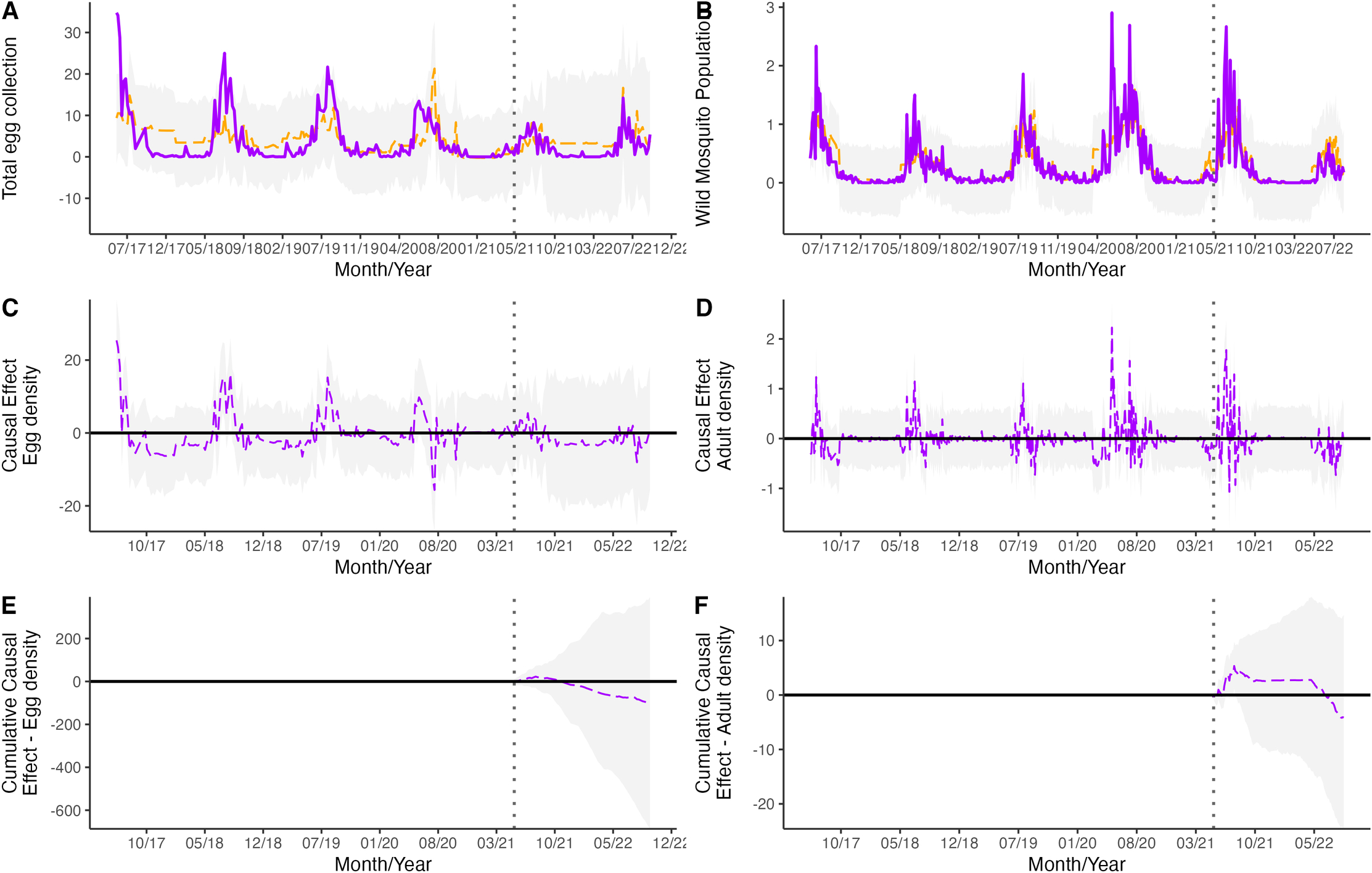

Supplement: S3 Fig — (TIF) [file pntd.0013256.s003.tif]

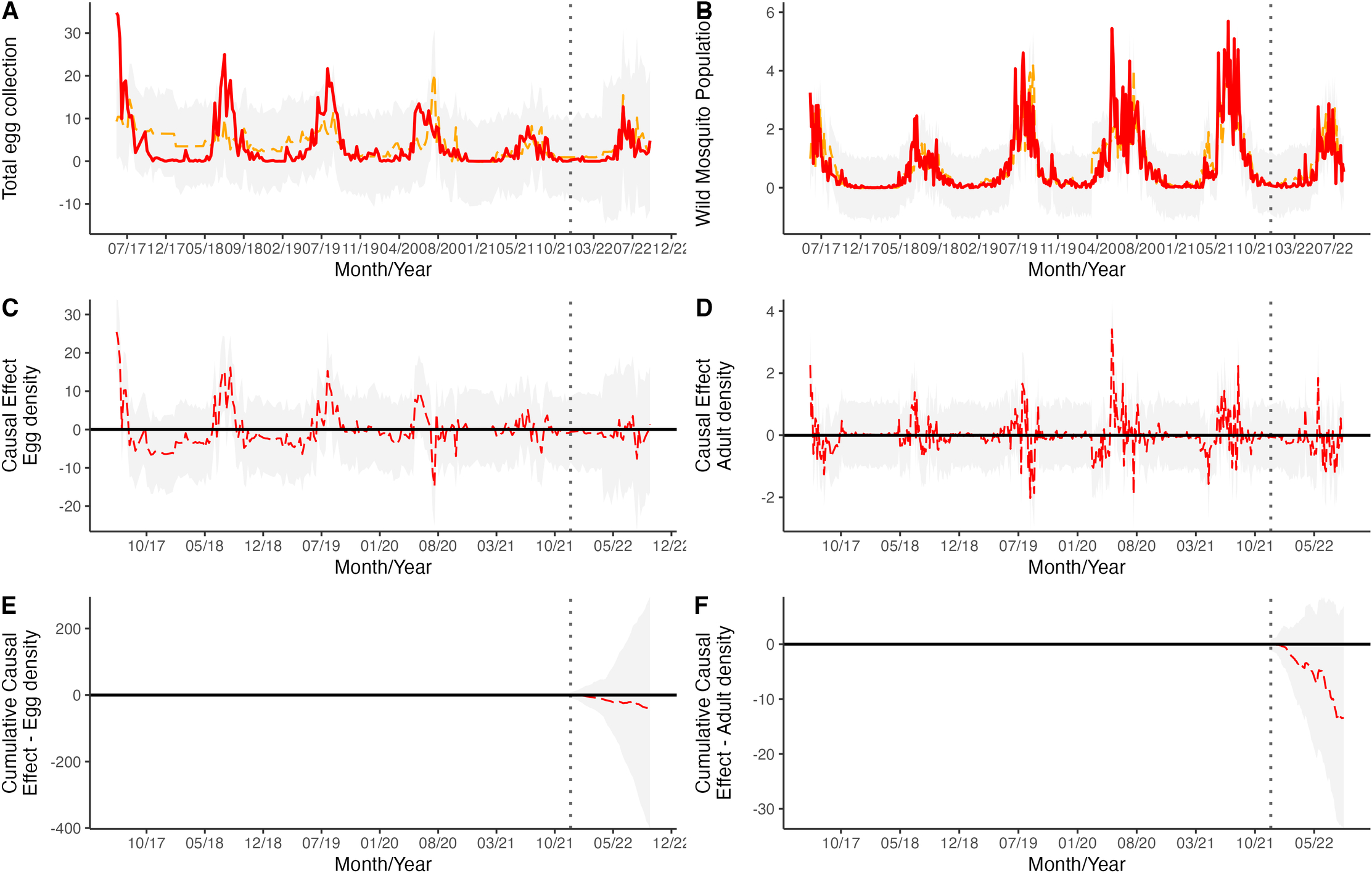

Supplement: S4 Fig — (TIF) [file pntd.0013256.s004.tif]
